# Cantharidin and sodium fluoride attenuate the negative inotropic effect of the A_1_-adenosine receptor agonist N^6^-(R)-phenylisopropyl adenosine in isolated human atria

**DOI:** 10.1007/s00210-024-03402-2

**Published:** 2024-08-30

**Authors:** R. Schwarz, B. Hofmann, U. Gergs, J. Neumann

**Affiliations:** 1 Medical Faculty, Institute for Pharmacology and Toxicology, Martin Luther University Halle-Wittenberg, Halle (Saale), Germany; 2 Cardiac Surgery, Medizinische Fakultät, Martin-Luther-Universität Halle-Wittenberg, 06097 Halle, Germany

**Keywords:** Cantharidin, Sodium fluoride, Adenosine receptor, Human atrium, Mouse atrium, Phosphatases

## Abstract

Cantharidin and sodium fluoride inhibit the activity of serine/threonine protein phosphatases 1 (PP1) and 2A (PP2A) and increase the force of contraction in human atrial preparations. R-phenylisopropyl adenosine (R-PIA) acts as an agonist at A_1_-adenosine receptors. R-PIA exerts a negative inotropic effect on human atria. The effect of R-PIA—and its various manifestations—are currently explained as a function of the inhibition of sarcolemmal adenylyl cyclase activity and/or opening of sarcolemmal potassium channels. We hypothesise that cantharidin and sodium fluoride may attenuate the negative inotropic effect of R-PIA. During open heart surgery, trabeculae carneae from the right atrium were obtained for human atrial preparations (HAPs). These trabeculae were mounted in organ baths and electrically stimulated at 1 Hz. Furthermore, we studied isolated electrically stimulated left atrial (LA) preparations from female wild-type mice (CD1). The force of contraction was recorded under isometric conditions. R-PIA (1 µM) exerted a rapid negative inotropic effect in the HAPs and mice LA preparations. These negative inotropic effects of R-PIA were attenuated by pre-incubation for 30 min with 100-µM cantharidin in HAPs, but not in mice LA preparations. Adenosine signals via A_1_ receptors in a species-specific pathway in mammalian atria. We postulate that R-PIA, at least in part, exerts a negative inotropic effect via activation of serine/threonine phosphatases in the human atrium.

## Introduction

**Fig. 1:**
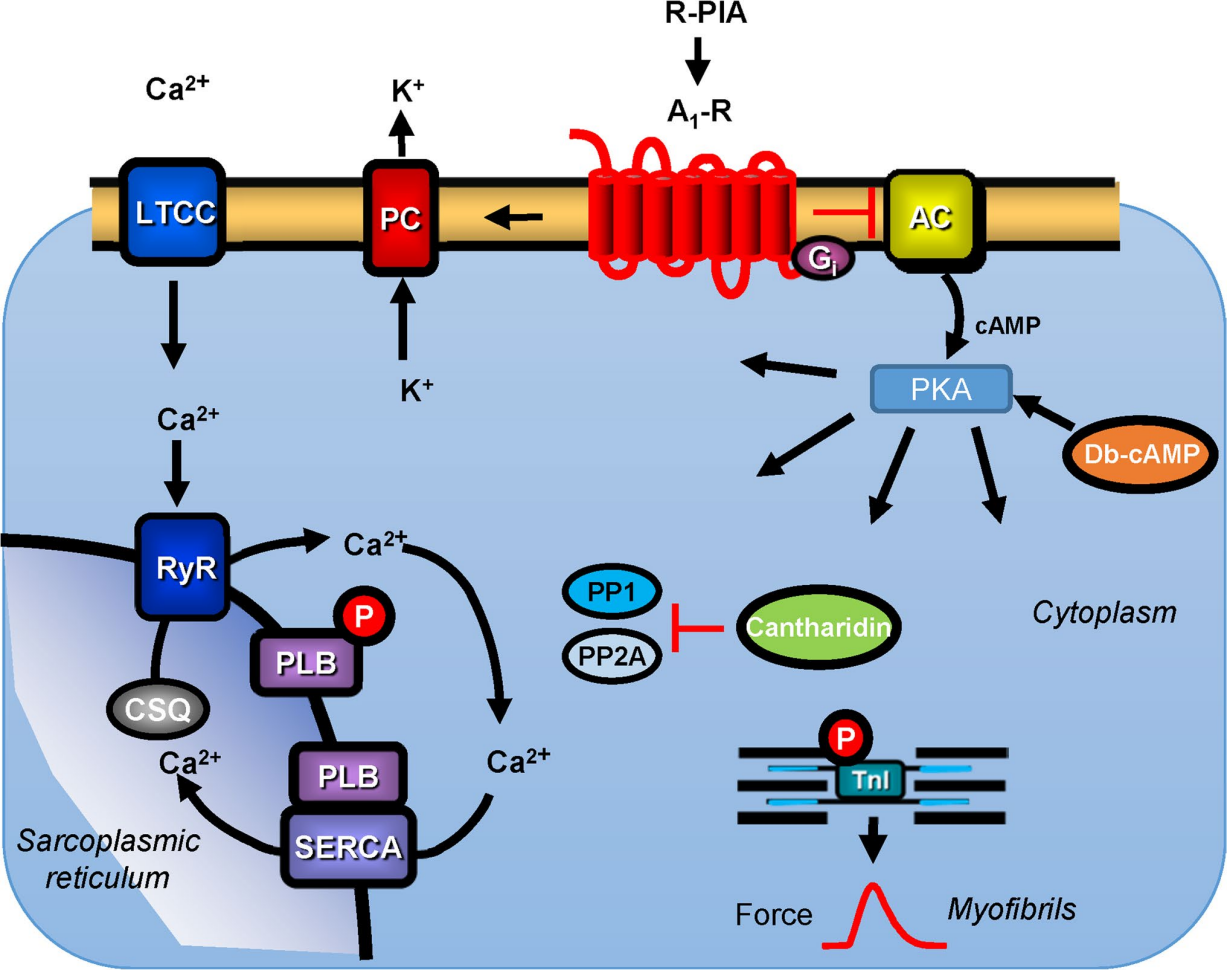
Scheme of a cardiomyocyte The Ca^2+^ for force generation in the heart is derived in part from *trigger* Ca^2+^ via L-type Ca^2+^ channels and, to a greater extent, via subsequent release of Ca^2+^ from the sarcoplasmic reticulum (SR) via ryanodine receptors (RYR). Cardiac relaxation is brought about via the phosphorylation of phospholamban (PLB) and the inhibitory subunit of troponin. Phosphorylated PLB de-inhibits the activity of the SR-Ca^2+−^ATPase (SERCA), and Ca^2+^ is rapidly moved from the cytosol into the SR, supporting relaxation. Furthermore, phosphorylation of the inhibitory subunit of troponin (TnI) reduces the Ca^2+^ sensitivity of myofilaments, hastening relaxation. Ca^2+^ can be released from the SR via ryanodine receptors. The activities of serine/threonine protein phosphatases (PP) PP1 and PP2A are inhibited by cantharidin or by sodium fluoride. R-PIA-stimulating A_1_-adenosine receptors may inhibit the enzymatic activity of adenylyl cyclases (AC) via a pertussis toxin-sensitive G-protein and/or may open potassium channels (PC), and/or may open the L-type Ca^2+^ channel (LTCC), and/or may directly or indirectly activate PP. AC can be inhibited by G_i_, an inhibitory guanosine triphosphate (GTP) binding protein, and can catalyse cAMP (3′,5′-cyclic adenosine monophosphate) formation. This cAMP activates cAMP-dependent protein kinases (PKA), which phosphorylates several cardiac proteins, such as PLB, RyR, LTCC and TnI, indicated by P in the circle

β-Adrenoceptors activate adenylyl cyclases via stimulatory guanosine triphosphate (GTP) binding proteins, thereby inducing the formation of 3′,5′-cyclic adenosine monophosphate (cAMP), which then stimulates cAMP-dependent protein kinase (PKA). PKA phosphorylates specific amino acids in regulatory proteins (serine and threonine) and thereby activates several proteins (Fig. 1), which are dephosphorylated by protein phosphatases (PPs) (Herzig and Neumann 2000; Neumann et al. 2021).

In human and animal cardiac preparations, both cantharidin and sodium fluoride inhibit PP1 and PP2A (Herzig and Neumann 2000). This inhibition is functionally relevant: cantharidin increases the force of contraction in guinea pig papillary muscle, mice atrial preparations and human atrial muscle and ventricular preparations by increasing the phosphorylation state of regulatory proteins (Neumann et al. 1995b; Schwarz et al. 2023a, b).

Furthermore, our research team, as well as other researchers, has supplied circumstantial evidence that the effect of R-phenylisopropyl adenosine (R-PIA) in reducing the force of contraction—at least concomitantly with β-adrenoceptor stimulation in the mammalian ventricle—involves not only inhibition of cAMP production but also activation of cardiac PPs (Herzig et al. 1995; Gupta et al. 2002).

In this study, we postulate that—in the human atrium—R-PIA acts, in part, by stimulating PP1 and/or PP2A (Fig. 1). Therefore, if we then inhibit PP1 and/or PP2A by means of cantharidin or sodium fluoride, the negative inotropic effect of R-PIA in the human atrium should be attenuated.

Similar studies on carbachol in the human heart (atrium or ventricle) have failed to touch on R-PIA. Nonetheless, R-PIA, like carbachol, can reduce the force of contraction in human atrial preparations in part via the same potassium channel. Interestingly, even in the presence of a phosphodiesterase inhibitor (IBMX), R-PIA has a negative inotropic effect devoid of a reduction in cAMP or cGMP (Böhm et al. 1994), and this prompted all our subsequent studies on cardiac PPs. Alternatively, one can directly increase intracellular cAMP by adding dibutyryl-cAMP. This produces a positive inotropic effect in, for example, Langendorff-perfused guinea pig hearts or isolated rat atrial preparations (Rockoff and Dobson 1980; West et al. 1986). However, when 5 mM dibutyryl-cAMP is added, the applied adenosine—presumably via A_1_-adenosine receptors—subsequently fails to reduce the force of contraction. This is typically interpreted as evidence against any involvement of PPs in the negative inotropic effect of R-PIA. However, as we show in this study, this may simply result from using a much too high concentration of dibutyryl-cAMP. Indeed, approximately 0.3 mM dibutyryl-cAMP is required to increase the force of contraction half-maximally in isolated human ventricular preparations (Neumann et al. 1999). Therefore, we tested the interaction between R-PIA and such a low concentration of dibutyryl-cAMP in the human atrium. To the best of our knowledge, this has not been studied previously.

Primarily, we test the following hypotheses: (1) cantharidin attenuates the negative inotropic effect of R-PIA in isolated mouse left atria and human atria and (2) sodium fluoride attenuates the negative inotropic effect of R-PIA in isolated mouse left atria and human atria.

## Materials and methods

### Mice

To avoid gender differences, we used only left atrial preparations from female mice aged 9–17 months. Only wild-type mice with a CD1 background were used in our experiments.

### Humans

Human specimens were obtained from male and female patients aged 60–82 years. The cardiac drug therapy of these patients included apixaban, furosemide, metoprolol and acetyl salicylic acid. This study was approved by a local ethical committee, and the patients from whom the human study specimens were obtained gave their written informed consent.

### Contractile studies in mice and humans

Our methods for atrial contraction experimentation studies on human samples have been published previously and were not altered in this study (Schwarz et al. 2023a, b). Right atrial preparations from humans and left atrial preparations (auricles) from wild-type mice were isolated and mounted in organ baths as described in a previous study (Neumann et al. 2003). The bathing solution—a modified Tyrode’s solution—in the organ baths contained NaCI (119.8 mM), KCI (5.4 mM), CaCl_2_ (1.8 mM), MgCl_2_ (1.05 mM), NaH_2_PO_4_ (0.42 mM), NaHCO_3_ (22.6 mM), Na_2_EDTA (0.05 mM), ascorbic acid (0.28 mM) and glucose (5.05 mM). The bathing solution was gassed continuously with 95% O_2_ and 5% CO_2_ and maintained at 37 ℃ and pH 7.4.

The specimens (human right atrial muscle strips and left atrial preparations from mice) were electrically stimulated at 1 Hz using a Grass Stimulator SD9 (Grass Instruments, Quincy, MA, USA), with 5-ms rectangular impulses of direct current at a 10% higher voltage than required for initiation of the heartbeat. The preparations were stretched carefully in such a way as to ensure that the maximum force (i.e., maximal difference between systolic and diastolic tensions) was generated which was about 4 mN. Force was measured under isometric conditions, and the signals were amplified with a bridge amplifier and fed into a personal computer. The hardware and software utilised were procured from AD Systems, Heidelberg, Australia, through their European outlet.

Drug application was performed as follows: After equilibration was reached, cantharidin (at 100 µM concentration) was added to the mouse left atrial preparations and human right atrial preparations. Then, where indicated, R-PIA was applied cumulatively or as a single concentration to the preparations.

Cantharidin (CANT, 100 mM in dissolved dimethyl sulfoxide (DMSO)) and (R)-N^6^-(1-methyl-2-phenylethyl)-adenosine (i.e., R-PIA) were purchased from Sigma-Aldrich (now Merck) in Taufkirchen, Germany. DMSO, at the concentrations used, slightly reduced the force of contraction, as reported in a previous study (Schwarz et al. 2023a). All other chemicals were of the highest purity grade commercially available. Deionised water was used throughout the experiments, and stock solutions were prepared fresh daily.

### Data analysis

The data are presented as the mean ± standard error of the mean. Statistical significance was estimated using analysis of variance, followed by Dunnett’s or Bonferroni’s *t* test. A *p* value of < 0.05 was considered significant.

## Results

### Cantharidin: mice atria

**Fig. 2:**
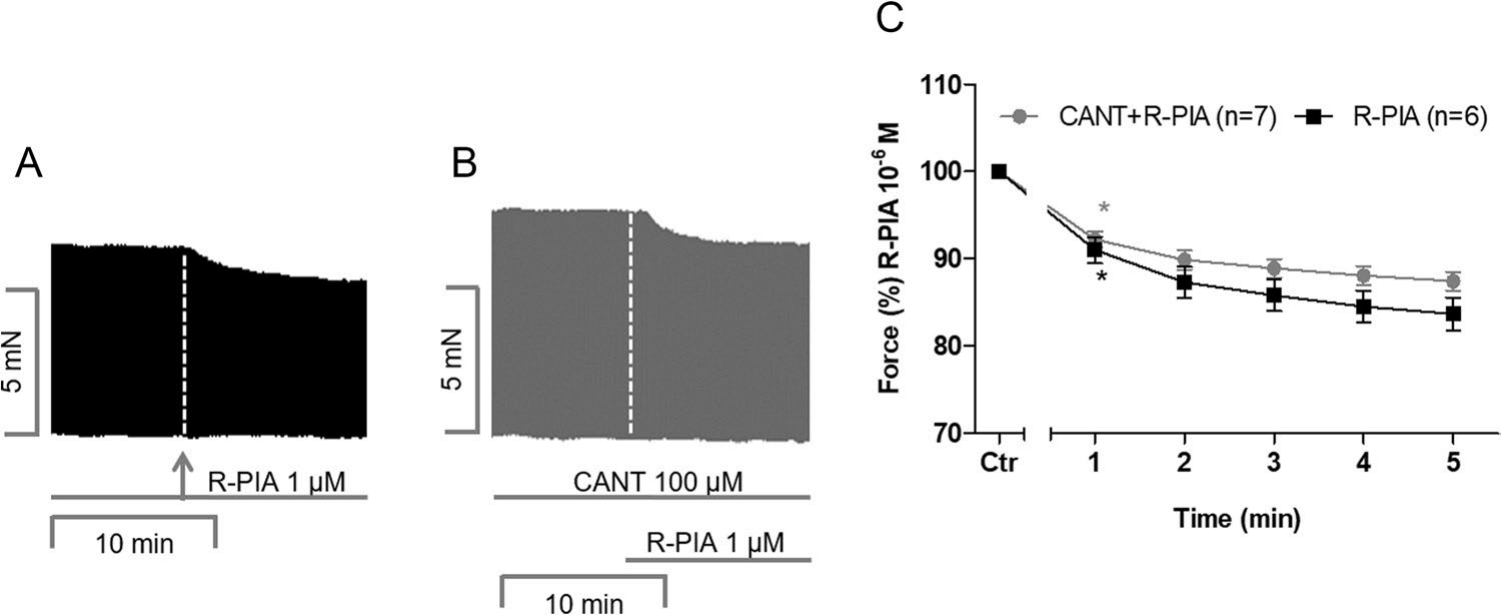
Impact of cantharidin on the negative inotropic effect of R-PIA in mouse left atrium. **A**, **B** Original recording of the effect of 1 µM R-PIA alone or in the presence of 100-µM cantharidin (CANT) on the force of contraction in milli Newton (mN, ordinate) over time in min (abscissa). **C** Time course of the negative inotropic effect of 1 µM R-PIA alone or in the presence of CANT (100 µM) in isolated electrically driven left atrial preparations from mice. The ordinate gives the force of contraction in % of pre-drug value before application of R-PIA, * first significant difference versus Ctr. The numbers in brackets indicate the number of experiments. Ctr indicates the value prior to the application of R-PIA

To electrically stimulate left atrial preparations from mice, 1 µM R-PIA was applied in the absence of CANT and in the presence of CANT. The original tracings are presented in Fig. 2A and B, respectively. The negative inotropic effect of R-PIA was not attenuated in the presence of CANT; the original recordings are presented in Fig. 2A and B, and the data on the force of contraction are summarised in Fig. 2C. We previously reported that CANT, at a 100 µM concentration, by itself increases the force of contraction in left atrial preparations from mice (Schwarz et al. 2023a, b). Under these conditions, R-PIA exerted a negative inotropic effect that was similar in the absence and presence of CANT (Fig. 2C).

### Cantharidin: human atria

**Fig. 3:**
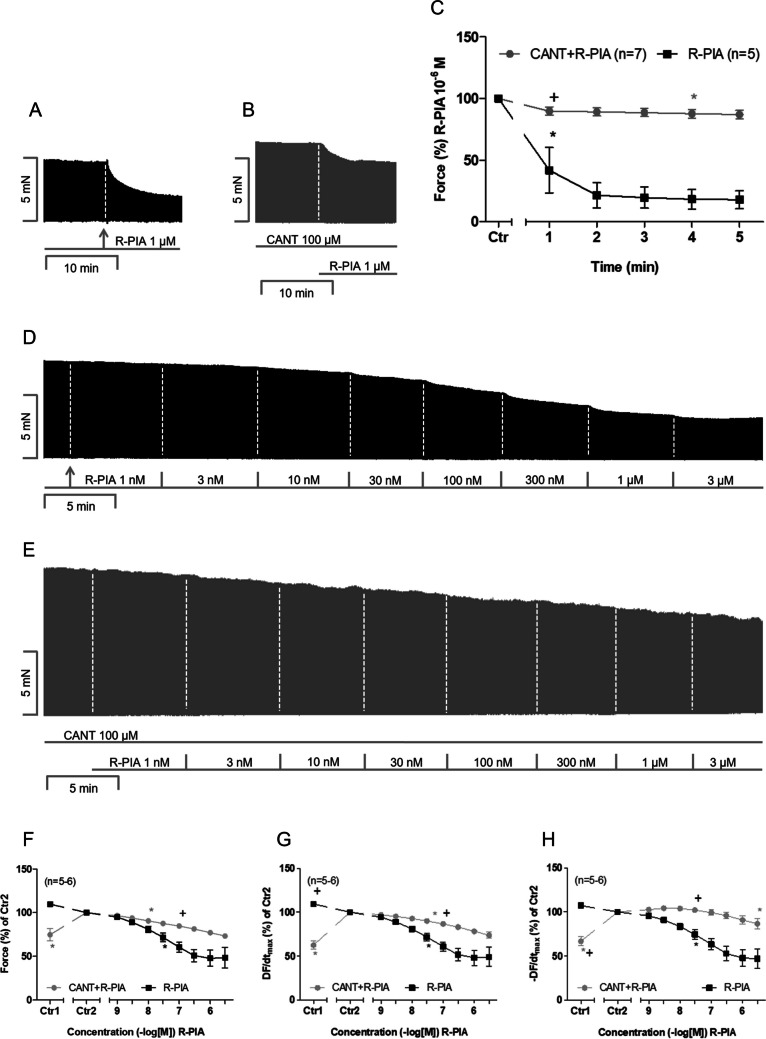
Cantharidin attenuates the negative inotropic effect of R-PIA on contractile parameters in human right atria. **A**, **B** Original recording of the effect of 1 µM R-PIA alone or with pre-applied cantharidin (100 µM) on the force of contraction in milli Newton (mN, ordinate) over time in min (abscissa). **C** Time course of the negative inotropic effect of 1 µM R-PIA alone or in the presence of CANT (100 µM) in isolated electrically driven human right atrial preparations. Ordinate gives the force of contraction in % of pre-drug value before application of R-PIA, * and + first significant difference versus Ctr or R-PIA in the presence of CANT, respectively. The numbers in brackets indicate the number of experiments. Ctr indicates the value prior to the application of R-PIA. **D**, **E** Original recording of the effect of cumulatively applied R-PIA alone or in the presence of 100-µM cantharidin on the force of contraction in milli Newton (mN, ordinate) over time in min (abscissa). **F** Concentration-dependent negative inotropic effect of cumulatively applied R-PIA alone or in the presence of CANT (100 µM) in isolated electrically driven human right atrial preparations. Ordinate gives the force of contraction in % of value before R-PIA (Ctr2). * and + first significant difference versus Ctr2 or R-PIA in the presence of CANT, respectively. The numbers in brackets indicate the number of experiments. Ctr1 indicates the pre-drug value. **G**, **H** Concentration-dependent effect of cumulatively applied R-PIA alone or in the presence of CANT (100 µM) on rate of tension development and on rate of tension relaxation in isolated electrically driven human right atrial preparations. Ordinate gives the rate of tension development (DF/dt_max_) or the rate of tension relaxation (− DF/dt_max_) in % of the value before R-PIA (Ctr2). * and + first significant differences versus Ctr2 or R-PIA in the presence of CANT, respectively. The numbers in brackets indicate the number of experiments. Ctr1 indicates the pre-drug value

For human atrial preparations, we used two different experimental set-ups. First, 100 µM CANT was utilised, and it raised the force of contraction (Ctr1 versus Ctr2 in Fig. 3F), which is consistent with the findings of previous studies (Schwarz et al. 2023a, b). Thereafter, R-PIA was applied noncumulatively or cumulatively. Notably, similar to the effect in mice atria, the effect of R-PIA in human atria is monophasic. This contrasts with carbachol, which initially induces a negative inotropic effect that is subsequently followed by a positive inotropic effect at higher concentrations of carbachol (Schwarz et al. 2023b), indicating that R-PIA stimulates only one type of receptor (i.e., A_1_-adenosine receptor), while carbachol stimulates two receptor types in the human heart, i.e., the M_2_- and M_3_-muscarinic receptors (Du et al. 1995). At a 1 µM concentration, R-PIA elicited a pronounced negative inotropic effect; the original recording is presented in Fig. 3A. However, the negative inotropic effect of 1 µM R-PIA was attenuated in the presence of CANT; the original recording is presented in Fig. 3B. The data on the negative inotropic effect of 1 µM R-PIA are summarised in Fig. 3C. Comparing Ctr1 and Ctr2 in Fig. 3C, the positive inotropic effect of CANT may be determined. Under the given conditions, CANT increased the rate of tension development and the rate of tension relaxation. Next, we cumulatively applied R-PIA to the specimens in the absence and presence of 100 µM CANT (Fig. 3D and E). Again, CANT attenuated the concentration-dependent negative inotropic effect of R-PIA; the data are summarised in Fig. 3F. Reductions in the rate of tension development and the time of relaxation effectuated by R-PIA were also attenuated by CANT (Fig. 3G and H).

### Sodium fluoride: human atria

**Fig. 4:**
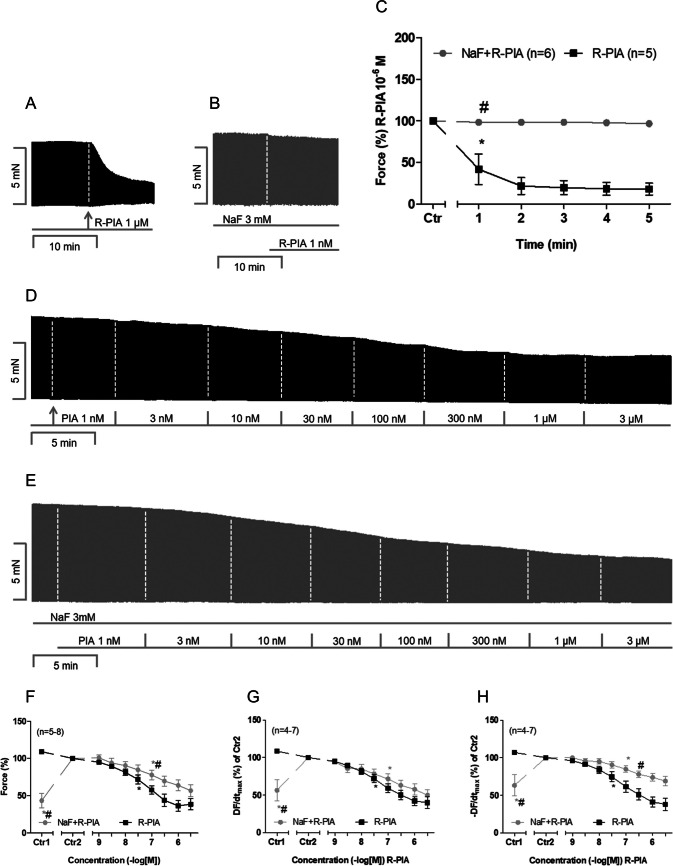
Sodium fluoride attenuates the negative inotropic effect of R-PIA on contractile parameters in the human right atrium **A**, **B** Original recording of the effect of 1 µM R-PIA alone or with pre-applied sodium fluoride on the force of contraction in milli Newton (mN, ordinate) over time in minutes in human right atrial preparations (min, abscissa). **C** Time course of the negative inotropic effect of 1 µM R-PIA alone or in the presence of sodium fluoride (3 mM) in isolated electrically driven human right atrial preparations. Ordinate gives the force of contraction in % of pre-drug value before the application of R-PIA, * and # first significant difference versus Ctr or R-PIA in the presence of sodium fluoride. The numbers in brackets indicate the number of experiments. Ctr indicates the value prior to the application of R-PIA. **D**, **E** Original recording of the effect of R-PIA (cumulatively applied) alone or in combination with 3 mM sodium fluoride (NaF) on the force of contraction in milli Newtons (mN, ordinate) over time in minutes in human right atrial preparations (min, abscissa). **F** Concentration-dependent negative inotropic effect of R-PIA alone or in the presence of sodium fluoride (3 mM) in isolated electrically driven human right atrial preparations. Ordinate gives the force of contraction in % of value before R-PIA (Ctr2). * and # first significant difference versus Ctr2 or R-PIA in the presence of sodium fluoride. The numbers in brackets indicate the number of experiments. Ctr1 indicates the pre-drug value. **G**, **H** Concentration-dependent effect of cumulatively applied R-PIA alone or with pre-applied sodium fluoride (3 mM) on the rate of tension development and on the rate of tension relaxation in isolated electrically driven human right atrial preparations. Ordinate gives the rate of tension development (DF/dt_max_) or the rate of tension relaxation (DF/dt_max_) in % of the value before R-PIA (Ctr2). * and # first significant differences versus Ctr2 or R-PIA in the presence of sodium fluoride, respectively. The numbers in brackets indicate the number of experiments. Ctr1 indicates the pre-drug value

For CANT in human atrial preparations, we used two experimental set-ups. Sodium fluoride (3 mM), similar to CANT, exerted a positive inotropic effect (Fig. 4F, Ctr1 versus Ctr2) in human atrial preparations, as has been reported previously (Schwarz et al. 2023b). The original recordings for the application of 1 µM R-PIA in the absence and presence of 3 mM sodium fluoride are presented in Fig. 4A and B. Similar to CANT, sodium fluoride attenuated the negative inotropic effect of R-PIA (Fig. 4C). Cumulatively applied R-PIA, in the absence of sodium fluoride, exerted a concentration-dependent negative inotropic effect over time (Fig. 4D). However, in the presence of sodium fluoride, the negative inotropic effect of R-PIA was attenuated (Fig. 4E). The data on R-PIA are summarised in Fig. 4F. Under these experimental conditions, R-PIA also reduced the rate of tension development (Fig. 4G) and the rate of tension relaxation (Fig. 4H). The effects of R-PIA on the rate of tension relaxation were attenuated by the addition of sodium fluoride, but the effects of R-PIA on the rate of tension development were unaffected (Fig. 4G and H).

### Dibutyryl-cAMP (Db-cAMP): human atria

**Fig. 5:**
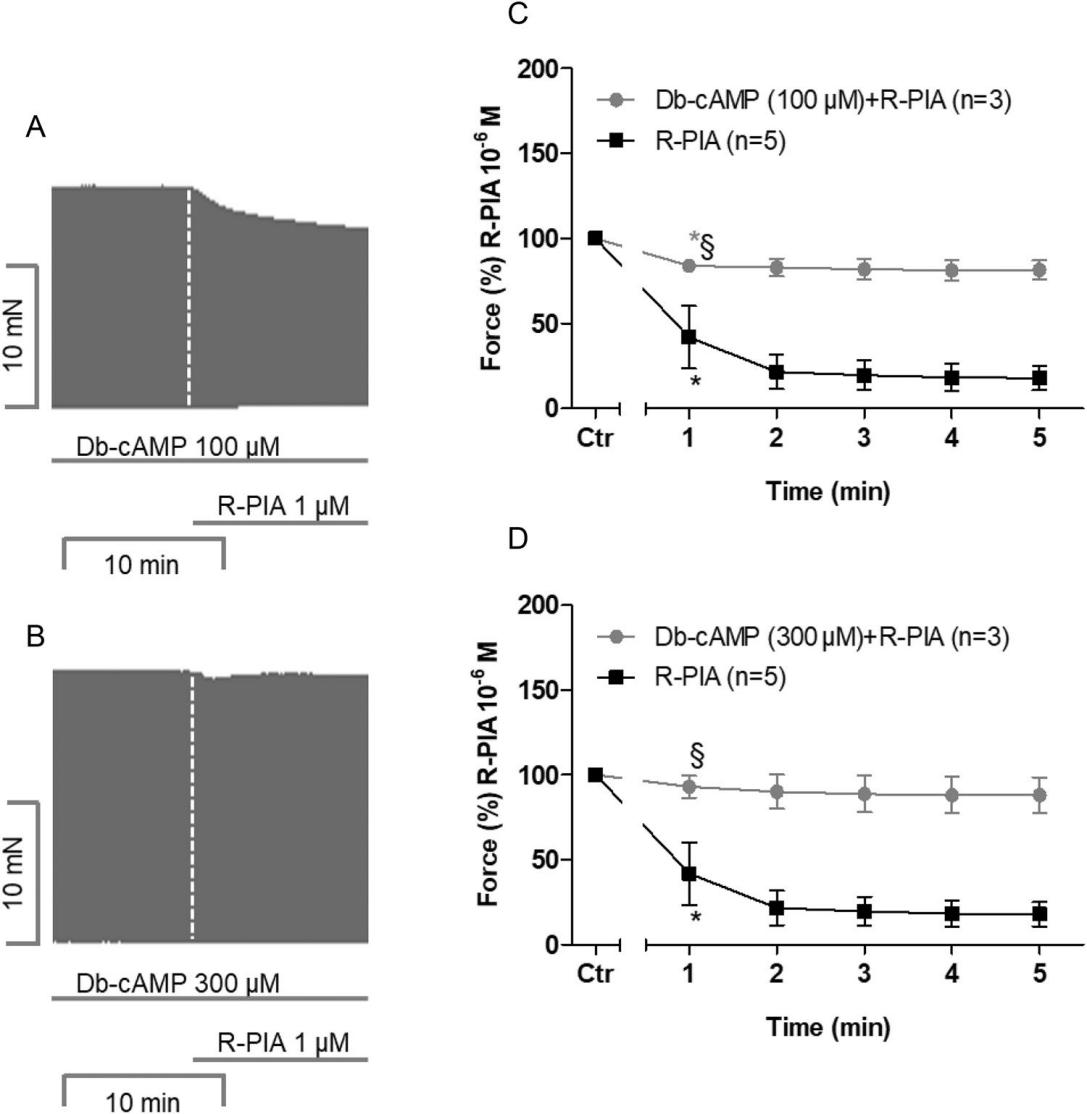
Dibutyryl-cAMP attenuates the negative inotropic effect of R-PIA on contractile parameters in human right atria. **A** Original recording of the effect of 1 µM R-PIA on the force of contraction in the presence of 100 µM dibutyryl-cAMP (Db-cAMP) in milli Newton (mN, ordinate) over time in minutes in human right atrial preparations (min, abscissa). **B** Original recording of the effect of 1 µM R-PIA on the force of contraction in the presence of 300 µM dibutyryl-cAMP (Db-cAMP) in milli Newton (mN, ordinate) over time in minutes in human right atrial preparations (min, abscissa). **C**, **D** Time course of the negative inotropic effect of 1 µM R-PIA alone or in the presence of 100 µM or 300 µM dibutyryl-cAMP in isolated electrically driven human right atrial preparations. Ordinate gives the force of contraction in % of pre-drug value before the application of R-PIA, * and § first significant difference versus Ctr or R-PIA in the presence of dibutyryl-cAMP, respectively. The numbers in brackets indicate the number of experiments. Ctr indicates the value prior to the application of R-PIA

We used dibutyryl-cAMP (Db-cAMP) as a stimulatory drug for PKA experimentation. This was done to intensify the force of contraction without any previous adenylyl cyclase stimulation (Fig. 1). In other words, R-PIA can reduce the force of contraction without any previous stimulation to induce cAMP generation in sarcolemma. Db-cAMP was applied to human atrial preparations at two different concentrations: 100 µM and 300 µM. The original recordings are presented in Fig. 5A and B. Both concentrations—with 300 µM being slightly more efficient—attenuated the negative inotropic effect of R-PIA (Fig. 5C and D).

## Discussion

In our view, the main new finding in this study is the observation that the PP inhibitors CANT and sodium fluoride attenuate the negative inotropic effect of R-PIA in the human atrium.

Specifically, the contractile effects of sodium fluoride are species- and region-dependent. For instance, we have observed that 3 mM sodium fluoride (NaF) has a positive inotropic effect on guinea pig papillary muscle (Neumann et al. 1995a; Neumann and Scholz 1998). In contrast, 3 mM NaF has been observed to decrease the force of contraction in guinea pig and rat atria until the preparations go into asystole (Neumann and Scholz 1998).

In guinea pig ventricular preparations and human ventricular preparations, R-PIA alone does not decrease the force of contraction (Borea et al. 2018). However, when force generation was pre-stimulated by isoprenaline (a β-adrenoceptor agonist) or 3-isobutyl-1-methylxanthine (an unselective phosphodiesterase inhibitor), R-PIA produced a negative inotropic effect in guinea pig papillary muscle that was almost completely neutralised when 3 mM NaF was applied (Neumann et al. 1995a).

In this study, we researched mouse and human atrial preparations. In mice atria and human atria, the direct negative inotropic effect of R-PIA was attenuated by previously applied NaF. However, we cannot be sure that NaF alone inhibits cardiac phosphatases. Indeed, NaF can interfere with mitochondrial function and G-proteins (Neumann and Scholz 1998). However, because CANT acts similarly, we postulate that NaF uses phosphatase inhibition to counteract the contractile effects of R-PIA.

An open question in this field—despite our past efforts and those of other researchers—is the question of how exactly cardiac A_1_-adenosine receptors couple to phosphatases. Based on our present data, it is uncertain whether genetic manipulation of mice will help answer this question, as mice and humans seem to signal differently via A_1_-adenosine receptors. We speculate that human cardiomyocytes derived from stem cells may be a more suitable model for further research in this direction. In such human cardiomyocytes, genetic manipulation might be worth the effort.

We have shown previously, at the mRNA and protein levels, that the catalytic subunits of PP1 and PP2A exist in the human atrium and mice atria (Lüss et al. 2000). Hence, the targets of CANT and NaF are expressed in the human atrium and mice atria (Lüss et al. 2000). The activity of PP1 and PP2A (measured together) was higher in the human heart than in the mouse heart (Neumann et al. 1997; Brüchert et al. 2008). This might explain why cantharidin was more potent to raise force of contraction in the mouse preparations than in the human preparations (Schwarz et al. 2023a). One might speculate that 50% of inhibition of PP is required to detect any positive inotropic effect of cantharidin. Hence, conceivably less cantharidin is required (because PP activity in lower) in mouse atrium compared to human atrium and thus less cantharidin is required in mouse than in human atrium. In addition, we cannot rule out that cell permeability differences exist for cantharidin between human heart and mouse hearts which might contribute to the higher potency of cantharidin to elicit a positive inotropic in the mouse atrium compared to the human atrium. Indeed, there is evidence in mouse liver for a membrane transport of cantharidin (Erdödi et al. 1995). Some additional species differences may lie in the accessory proteins that assemble with the catalytic subunits of PP. Our data on dibutyryl-cAMP seem to indicate that there is room for the effect of PPs. When using a small concentration of dibutyryl-cAMP (100 µM or 300 µM) that does not maximally stimulate phosphorylation, R-PIA, presumably via the activation of distally located PPs (Fig. 1), can reduce force generation. This is circumstantial evidence, as the inhibition of adenylyl cyclases is not necessary for the negative inotropic effect of R-PIA because dibutyryl-cAMP is not known to stimulate cardiac adenylyl cyclases.

The cardiac action of R-PIA differs between species and across cardiac regions (i.e., the atrium and the ventricle). In the atrium, the functionally negative inotropic effect of R-PIA is well established in human atria and mice atria (Böhm et al. 1994; Boknik et al. 2009; Du et al. 1994; Neumann et al. 2003). The negative inotropic effect of R-PIA vanishes in A_1_-adenosine receptor knockout mice and is thus mediated by A_1_-adenosine receptors (Burnstock 2017). Similar findings have been reported in human atrial samples. In this regard, studies on receptor-specific antagonists have arrived at the same conclusion: the effect of R-PIA is blocked by DPCPX, thus confirming that the effect is mediated by A_1_-adenosine receptors (Böhm et al. 1994). Thus, on the one hand, the negative inotropic effect of R-PIA in mice and human atria is probably mediated by A_1_-adenosine receptors. On the other hand, we have repeatedly shown that the negative inotropic effect of R-PIA in the presence of isoprenaline in the guinea pig ventricle involves phosphorylation of phosphatase inhibitor-1 and subsequent inhibition of PP1 (Gupta et al. 1993, 1994, 2002; Neumann et al. 1994). Other studies have presented evidence that in the ventricle, R-PIA can also activate PP2A (Liu and Hofmann 2002). Hence, there is precedence for R-PIA activating PP1 or PP2A in the mammalian heart. In this study, we postulate that the same occurs in the human atrium. R-PIA activates PP1 or PP2A in the human atrium, but this is inhibited by CANT and sodium fluoride. The positive inotropic effect of cantharidin in the human atrial preparations is accompanied by an increase the phosphorylation state of regulatory proteins like phospholamban (Schwarz et al. 2023a). We propose that the remaining negative inotropic effect of R-PIA in the perpetual presence of CANT is due to the activation of potassium ion channels and the subsequent inhibition of calcium ion channels in the atrium in a phosphorylation-independent fashion (Burnstock 2017).

This study has several limitations. We cannot offer an outline of the biochemical chain of events from the receptor to the phosphatase in the human atrium. There are data that acetylcholine can activate PPs in cardiac membranes after perfusion of whole guinea pig hearts (Ahmad et al. 1989). Similar studies in mouse heart with R-PIA are, to the best of our knowledge, lacking and would better define signal transduction pathways. However, it is known that NaF and CANT inhibit phosphatase activity in human and mouse hearts (Neumann et al. 1994, 1995a). Moreover, we have not measured phosphatase activity in extracts from HAPs and extracts from mouse LA. Moreover, CANT and dibutyryl-cAMP in human muscle strips increase the phosphorylation state of phospholamban, for example (Schwarz et al. 2023b; Neumann et al. 1999). At least in guinea pig ventricular cardiomyocytes, acetylcholine reduced the phosphorylation of phospholamban that had been stimulated by dibutyryl-cAMP (Gupta et al. 1998). These animal data suggest that a similar mechanism for dibutyryl-cAMP an R-PIA might exist in the human atrium. Moreover, in atrial cardiac preparations, A_1_-adenosine receptors can open potassium channels, and this leads to a shortening of the duration of the action potential and thus less time is available for Ca^2+^ entering the cardiomyocytes leading to decline in force of contraction in the atrium. To what extent any action of R-PIA on potassium channels in human atrium is relevant and is regulated by phosphatases needs to be elucidated. Moreover, the effect of R-PIA was more prominent in single application compared to cumulative application in the presence of cantharidin or NaF. One might speculate that in cumulative application, a receptor desensitisation occurs or coupling to additional G-proteins might take place for which there is some experimental evidence (Boknik et al. 2009). Moreover, basal force was different between patients and this might bias our findings.

In summary, we can now confirm at least in part the hypotheses formulated in the introduction: CANT and NaF attenuate the negative inotropic effect of R-PIA in human right atria but not in mice left atria. We speculate that CANT and NaF, in part, inhibit cardiac phosphatases stimulated by R-PIA—at least in the human right atrium. However, other additional mechanism cannot be ruled out but many contribute to the negative inotropic effects of R-PIA in HAP.

## Data Availability

No datasets were generated or analysed during the current study.
